# Ultrasonographic observation of the healing process in the gap after a Ponseti-type Achilles tenotomy for idiopathic congenital clubfoot at two-year follow-up

**DOI:** 10.1007/s00776-012-0312-y

**Published:** 2012-09-28

**Authors:** Hisateru Niki, Hiroshi Nakajima, Takaaki Hirano, Hirokazu Okada, Moroe Beppu

**Affiliations:** Department of Orthopaedic Surgery, St. Marianna University School of Medicine, 2-16-1 Sugao, Miyamae-ku, Kawasaki, Kanagawa 216-8511 Japan

## Abstract

**Background:**

Ponseti management usually requires Achilles tenotomy during the final stage of serial casting. However, we lack a good understanding of the sequential tendon healing process after tenotomy in the Ponseti bracing protocol. The purpose of this study was to clarify the ultrasonographic process of tendon healing in the gap for up to two years after Ponseti-type Achilles tenotomy in patients with clubfeet.

**Methods:**

We conducted an ultrasonographic study to clarify the sequential changes in gap healing for up to two years after tenotomy. The subjects were 23 patients with 33 clubfeet. Achilles tenotomy was performed at mean 10.4 (8–16) weeks after birth. Dynamic and static ultrasonography was performed before tenotomy and at 1, 2, 3, 4, 6, 8, and 12 weeks as well as at 4, 6, 12, 18, and 24 months after tenotomy.

**Results:**

Continuity and gliding were noted within four weeks. The united portion continued to thicken for up to three months after tenotomy. Starting from the fourth month, the healed portion began to lose its thickness, and this process continued into the sixth month. At one year, the thickness of the tendon did not differ much from that of the tendon on the opposing foot. In cases where patients had clubfoot on both feet and underwent simultaneous tenotomies, measurement of the tendons could not be accurately compared. At two years after tenotomy, slight irregularity of the internal structure persisted when compared with the unaffected foot. In addition, clinical and X-ray findings were evaluated simultaneously, and no recurrence was confirmed.

**Conclusions:**

To our knowledge, our results are the first to describe the process of gap healing in the tendon after tenotomy up to and beyond two years, as recommended in the Ponseti bracing protocol.

* Level of evidence* IV.

## Introduction

The Ponseti method is a well-established treatment for idiopathic congenital clubfoot deformity [1, 2]. The success rate of the Ponseti method has been >90 % [1, 3]. The Ponseti method consists of three phases; manipulation followed by weekly serial casting as the first phase, Achilles tenotomy and post-tenotomy casting as the second phase, and a bracing protocol as the third phase [2].

An Achilles tenotomy is often needed to correct a residual equinus deformity [2]. An Achilles tenotomy is a simple and safe technique with low invasiveness. In addition, there is no stiffness of the foot after tenotomy when compared with other traditional extensive soft-tissue releases [4]. Several studies found that 85 % of Achilles tenotomies were performed by the Ponseti method [1, 5–8]. This suggests the importance of the Achilles tenotomy in the Ponseti method.

Ponseti and others have described that a Ponseti-type Achilles tenotomy creates a gap that heals, eventually leaving the tendon in continuity [2, 9]. They have also observed clinically that the continuity of the Achilles tendon is restored within three weeks. Recently, so-called gap healing in divided tendons has been described in several studies. Four ultrasonographic studies of gap healing after Achilles tenotomy have been published [10–13]. However, those studies continued for only 6 weeks [10], 12 weeks [13], 6 months [11], and 1 year [12] post-tenotomy. In addition, changes in the structure of the healing tendon were not described fully up to complete tendon healing [10–13]. To our knowledge, there have been no studies which have described gap healing after Achilles tenotomy for up to two years, which is generally recommended in the Ponseti bracing protocol.

The purpose of this study was to clarify the ultrasonographic process of tendon healing in the gap for up to two years after Ponseti-type Achilles tenotomy in patients with clubfeet.

## Materials and methods

This investigation was approved by the Institutional Review Board of the St. Marianna University School of Medicine under an expedited review with a waiver of informed consent.

The subjects were 23 patients with 33 clubfeet. There were 14 males and 9 females. Inclusion criteria were (1) children with idiopathic congenital clubfoot treated with the Ponseti method; (2) the primary treatment had been performed at our institution; and (3) a minimum follow-up of two years. Exclusion criteria were associated follow-up treatments that were performed at other institutions, malformations, syndromic cases, neurologic disorders, and parental noncompliance.

Achilles tenotomy was performed following weekly serial casting. Casts were exchanged 6.1 times (mean; range 5–10 times) before tenotomy, and tenotomy was performed 10.4 weeks (mean; range 8–16) weeks after birth.

Children were placed on the exam table in the prone position. An ultrasound scan was performed over the posterior aspect of the Achilles tendon. Dynamic and static ultrasonography was performed by a single musculoskeletal imaging specialist (H.N.) before tenotomy and at 1, 2, 3, 4, 6, 8, and 12 weeks as well as at 4, 6, 12, 18, and 24 months after tenotomy. An Aloka SSD-650 scanner (Aloka, Co., Ltd., Tokyo, Japan) with a 10 MHz probe was used longitudinally to observe the characteristics of the tendon healing process after tenotomy. Both Achilles tendons were scanned, even in unilateral deformities, and the unaffected feet from the same patients were used as controls. For each tendon, continuity, gliding, and thickness were described.

The amount of deformity was clinically assessed according to the modified Catterall/Pirani scoring system [14] at the start of treatment, before tenotomy, and at the final follow-up examination.

Lateral radiographs with the foot in maximal dorsiflexion at the ankle and anteroposterior radiographs of the foot were obtained before tenotomy and at the time of the final follow-up examination.

### Surgical procedure and post-tenotomy management

The Achilles tenotomy was performed with general anesthesia as an inpatient procedure under the supervision of a consultant orthopedic surgeon. Incisions were made approximately 1 cm proximal to the tendon insertion with a Beaver blade, with the blade moving from medial to lateral and from deep to superficial. In each foot, clinical evidence of a successful tenotomy included a definitive increase in ankle dorsiflexion with a corresponding palpable gap between the ends of the divided tendon. After tenotomy, an above-the-knee molded cast, with the foot in abduction and dorsiflexion, was applied for three weeks. The cast was changed every week for ultrasonography. According to Ponseti’s protocol, an abduction foot brace was applied immediately after the last cast was removed. In our institution, an abduction foot brace was used full time for three months after the last cast was removed, and at nights and during naps for up to four years.

Further clinical and ultrasonographic assessment was undertaken during the study protocol. Imaging analysis was carried out by consensus between one musculoskeletal imaging specialist (H.N.) and one senior pediatric orthopedic surgeon (H.N.).

### Statistical methods

The paired *t* test was used to compare the X-ray parameters preoperatively and postoperatively. The level of significance was set at *p* < 0.05.

## Results

No complications were encountered following tenotomy. No patient suffered from delayed or incomplete healing or rupture.

### Ultrasonographic findings

After tenotomy, the gap was measured by ultrasonography, and the mean length of the gap was 11 (8–13) mm.

**Fig. 1:**
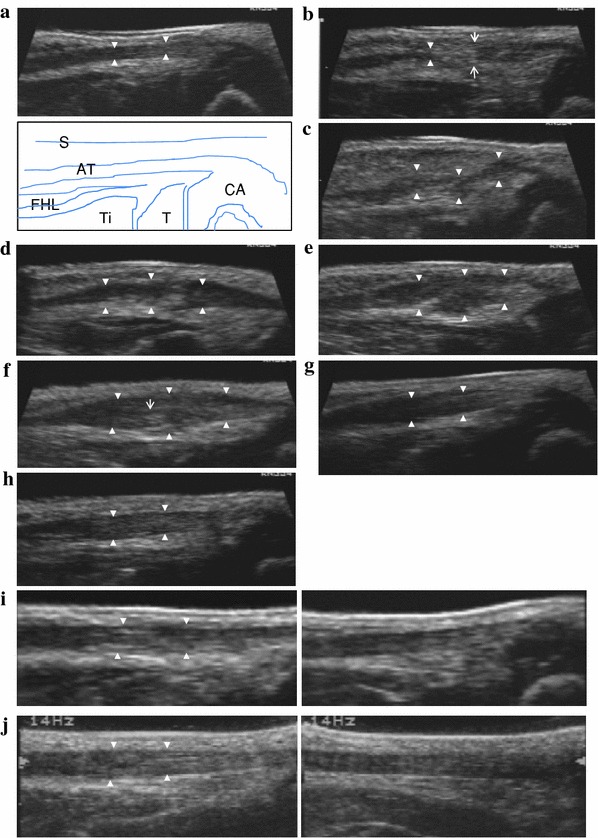
**a** Before tenotomy. Case 1 at two and a half months after birth. The Achilles tendon is clearly depicted as a low-echo region (*solid white triangles*). *AT* Achilles tendon, *CA* calcaneus, *T* talus, *Ti* tibia, *FHL* flexor hallucis longus, *S* skin. **b** 1–2 weeks after tenotomy. Case 1 at three months after birth and at 14 days after tenotomy. *Solid white triangles* show the proximal edge. *White arrows* show tenotomy portion. The gap zone is of mixed echogenicity. **c** 2–3 weeks after tenotomy. Case 1 at three months and one week after birth and at 21 days after tenotomy. *Solid white triangles* show that the lesion continued to be visible as a low echo. The gap is filled with irregular hypoechoic tissue. **d** 3–4 weeks after tenotomy. Case 1 at three and a half months after birth and at 28 days after tenotomy. Continuity of the tendon is identified. A bulbous appearance of the gap after tenotomy can be seen. **e** 4–6 weeks after tenotomy. Case 1 at three months and three weeks after birth and at 35 days after tenotomy. The united portion (*solid white triangles*) appears swollen. **f** 8–12 weeks after tenotomy. Case 1 at five months after birth and at 70 days after tenotomy. The united portion (*solid white triangles*) continues to thicken up to 8–12 weeks after tenotomy. The fibers are seen to display a linear configuration (*narrow white arrow*). **g** 14–16 weeks after tenotomy. Case 1 at six and a half months after birth and at 112 days after tenotomy. The healed portion (*solid white triangles*) begins to decrease in size. **h** Six months after tenotomy. Case 1 at eight and a half months after birth and at 169 days after tenotomy. The decease in size continued into the sixth month. Homogeneous tendon fibers are observed within the gap (*white solid triangles*). **i** One year after tenotomy. Case 1 at one year and two months after birth and at 358 days after tenotomy. *Left* shows the affected side and *right* the nonaffected side in the same case. The thickness does not differ much from that on the nonaffected side. The arrangement of tendon fibers within the gap is not similar at all to that on the normal side. **j** Two years after tenotomy. Case 1 at two years and three months after birth and at 719 days after tenotomy. *Left* shows the affected side and *right* the nonaffected side in the same case. Slight irregularity in the internal structure persists when compared with the nonaffected side

The following ultrasonographic findings were observed in all cases (see also Fig. 1a–j):

### Before tenotomy

The Achilles tendon was clearly depicted as a low-echo region (Fig. 1a).

### 1–2 weeks after tenotomy

The proximal edge could not dorsiflex at the ankle. The gap zone was of mixed echogenicity (Fig. 1b).

### 2–3 weeks after tenotomy

The lesion continued to show as a low echo. The gap was filled with irregular hypoechoic tissue, and in 14 (43 %) of 33 feet, the gap did not separate, even when the ankle was dorsiflexed gently and the tendon was seen gliding, indicating continuity (Fig. 1c).

### 3–4 weeks after tenotomy

Continuity and gliding of the tendon were noted in all cases. Occasionally, randomly arranged fibers were observed within the gap zone, and there was a characteristic increase in the diameter of the regenerating tendon in both the sagittal and coronal planes, resulting in a bulbous appearance (Fig. 1d).

### 4–6 weeks after tenotomy

The united portion was swollen, with the fibers arranged in a random manner, and the gap zone was still discernible (Fig. 1e).

### 8–12 weeks after tenotomy

The united portion continued to thicken for up to 8–12 weeks after tenotomy and was depicted as a low-echo region. Fibers were more frequently seen to be bridging the gap but were fewer in number and less densely packed than those in a normal tendon. The fibers were also seen to form in a more linear configuration (Fig. 1f).

### 14–16 weeks after tenotomy

Starting from the fourth month, the swelling in the healed portion began to decrease and continued to decrease into the sixth month. Comparing the thickness of the tendon and its changes over time between the affected and nonaffected sides in 13 unilateral cases, the affected side was an average of 2.8 times larger than the nonaffected side at 14–16 weeks after tenotomy. At six months after tenotomy, the affected side was an average of 1.2 times larger than the nonaffected side. The fibers of the united portion showed a definite linear configuration (Fig. 1g).

### Six months after tenotomy

The healing process of the gap had progressed further by the sixth month. Homogeneous tendon fibers were observed forming within the gap. However, the arrangement of tendon fibers was dissimilar to that in the normal tendon (Fig. 1h).

### One year after tenotomy

The thickness did not differ much from that on the nonaffected side. However, the arrangement of tendon fibers within the gap was dissimilar to that in the nonaffected side (Fig. 1i).

### Two years after tenotomy

The thickness continued to decrease, and slight irregularity of the internal structure persisted when compared with the nonaffected side (Fig. 1j).

### Clinical and X-ray findings

**Fig. 2:**
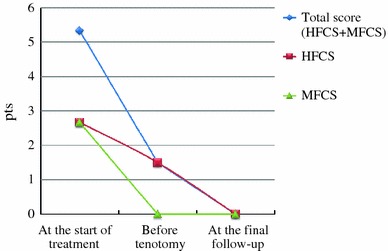
Modified Catterall/Pirani scores. *HFCS* hindfoot contracture, *MFCS* midfoot contracture. Total score (HFCS + MFCS): 0–6 pts, HFCS: 0–3 pts, MFCS: 0–3pts. Most abnormal: 6 pts, normal: 0 pts

Modified Catterall/Pirani scores obtained at the start of treatment, before tenotomy, and at the final follow-up examination are shown in Fig. 2.

The only X-ray parameter that changed significantly after tenotomy was the lateral tibiocalcaneal angle (LTiC) (*p* < 0.0001). The mean improvement in the LTiC after tenotomy was 25.8°. The anteroposterior and lateral talocalcaneal angles were not influenced significantly by Achilles tenotomy.

## Discussion

Ponseti and others clinically observed that the continuity of the Achilles tendon is restored in three weeks [1, 2, 4], and their finding was confirmed by our study. Additionally, we believe that it is important to discuss the gap-healing processes that occur after tenotomies have been performed on subjects under four months of age because the influence of age on the healing process and healing velocity should be considered, as well as to document the healing process for up to two years after tenotomy because slight irregularity of the internal structure persisted when compared with the nonaffected side at two years after tenotomy. To our knowledge, our results are the first to satisfy those two requirements, and to describe the process of gap healing in the tendon after tenotomy up to and beyond two years, as recommended in the Ponseti bracing protocol.

Healing of the tendon has been classically described as involving extrinsic or intrinsic factors or a combination of the two. In an extrinsic process, the gap is invaded by fibroblasts, and the repair leads to the formation of a fibrous scar with adhesions that favor the blood supply [15] but may interfere with the sliding mechanism, and may also represent a mechanical shortcoming [16, 17]. In an intrinsic repair process, a cascade of cellular and biochemical events takes place; fibroblast migration tends to proceed in an orderly fashion, and thus tendon tissue is regenerated in a fetal-like process [17]. Recent research has suggested that embryonic mechanisms may be responsible for healing in the adult tendon [16, 17]. The results of our study showed that the gap heals spontaneously and rapidly within three weeks after tenotomy. We provided objective evidence that a lesion that is still separated heals satisfactorily, and that three weeks is sufficient for a post-tenotomy cast. Knowing this will prevent repeated shortening or excessive prolongation of the time required for immobilization. Thus, the rapid healing seen in infants, in whom progenitor cells are abundant, may involve the same mechanism [10], although the predominance of either one of these processes depends on the tendon, grade of injury, blood supply, gap size, age, and mechanical factors [16, 17].

On the other hand, especially during the first six months, we found progressive maturation of the repair tissue, as evidenced by increasingly normal echogenicity and a fibrillar aspect of the parallel linear echotexture. Later on, this appearance became more patent, leading to the conclusion that a predominantly intrinsic mechanism is responsible for the formation of a normal or near-normal tendon. Ponseti’s long-term results indicate that divided human Achilles tendons display a normal surface anatomy with no adhesions and no tendency to rupture, suggesting that complete tendon recovery does take place [2]. However, our ultrasonographic findings indicated that although thickness recovered, slight irregularity of the internal structure persisted when compared with the nonaffected side, even two years after tenotomy. To our knowledge, our result is the first such description of the process of gap healing in the tendon for up to two years after tenotomy.

Our study was based on morphologic and dynamic changes. A weakness of the study is the fact that the measurements we obtained were not precise because the subjects were infants and were constantly moving. In this study, as all subjects were newborn infants under four months old, it was extremely difficult to define the end of the stump and measure the change in thickness over time, even though an observer (N.H.) was versed in ultrasonographic examination. Consequently, no numerical examination was conducted in this study due to the lack of accuracy, and only ultrasonographic observations of the gap healing process were conducted. Future studies should examine the influence of Achilles tenotomy on calf muscle atrophy.

In conclusion, sequential changes in Achilles tendon healing for up to two years after tenotomy were observed ultrasonographically. Gliding was noted within four weeks. At one year, the thickness of the tendon did not differ much from that of the normal tendon. At two years after tenotomy, slight irregularity of the internal structure persisted when compared with the nonaffected side.

## References

[1] Herzenberg JE, Radler C, Bor N (2002). Ponseti versus traditional methods of casting for idiopathic clubfoot. J Pediatr Orthop.

[2] Ponseti IV (1996). Congenital clubfoot: fundamentals of treatment.

[3] Colburn M, Williams M (2003). Evaluation of the treatment of idiopathic clubfoot by using the Ponseti method. J Foot Ankle Surg.

[4] Scher DM, Feldman DS, van Bosse HJ, Sala DA, Lehman WB (2004). Predicting the need for tenotomy in the Ponseti method for correction of clubfoot. J Pediatr Orthop.

[5] Morcuende JA, Abbasi D, Dolan LA, Ponseti IV (2005). Results of an accelerated Ponseti protocol for clubfoot. J Pediatr Orthop.

[6] Bor N, Herzenberg JE, Frick AL (2006). Ponseti management of clubfoot in older infants. Clin Orthop Relat Res.

[7] Bor N, Coplan JA, Herzenberg JE (2009). Ponseti treatment for idiopathic clubfoot. Minimum 5-year followup. Clin Orthop Relat Res.

[8] Dobbs MB, Rudzki JR, Purcell DB, Walton T, Porter KR, Gurnett CA. Factors predictive of outcome after use of the Ponseti method for the treatment of idiopathic clubfeet. J Bone Joint Surg (Am). 2004;86:22–7. 10.2106/00004623-200401000-00005 14711941

[9] de Gheledere A, Docquier PL (2008). Analytical radiography of clubfoot after tenotomy. J Pediatr Orthop.

[10] Baker SL, Lavy CB. Correction of clinical and ultrasonographic findings after Achilles tenotomy in idiopathic club foot. J Bone Joint Surg (Br). 2006;88:377–9. 10.1302/0301-620X.88B3.17273 16498015

[11] Niki H, Watanabe G, Hirano T, Tanaka T, Okada H, Beppu M, Nakajima H. Ultrasonographic observation of the healing process after Achilles tenotomy in congenital clubfoot: a preliminary report. J Jpn Soc Surg Foot. 2008;29(2):104–8 (in Japanese).

[12] Maranho DA, Nougueir-Barbosa MH, Simao MN, Volpon JB (2009). Ultrasonographic evaluation of Achilles tendon repair after percutaneous sectioning for the correction of congenital clubfoot residual equines. J Pediatr Orthop.

[13] Mangat KS, Kanwar T, Johnson K, Korah G, Prem H. Ultrasonographic phases in gap healing following Ponseti-type Achilles tenotomy. J Bone Joint Surg (Am). 2010;92:1462–7. 10.2106/JBJS.I.00188 20516322

[14] Lehman WB, Mohaideen A, Madan S, Scher DM, Van Bosse HJ, Iannacone M, Bazzi JS, Feldman DS. A method for the early evaluation of the Ponseti (Iowa) technique for the treatment of idiopathic clubfoot. J Pediatr Orthop (B). 2003;12:133–40. 10.1097/01.bpb.0000049579.53117.4a 12584499

[15] Potenza AD. Critical evaluation of flexor-tendon healing and adhesion formation within artificial digital sheaths. J Bone Joint Surg (Am). 1963;45:1217–33. 14077985

[16] Lin TW, Cardenas L, Soslowsky LJ (2004). Biomechanics of tendon injury and repair. J Biomech.

[17] Ingraham JM, Hauck RM, Ehrlich HP (2003). Is the tendon embryogenesis process resurrected during tendon healing?. Plast Reconstr Surg.

